# Supplementary material for: Safety and Feasibility Assessment of Repetitive Vascular Occlusion Stimulus (RVOS) Application to Multi-Organ Failure Critically Ill Patients: A Pilot Randomised Controlled Trial
Source: J Clin Med. 2022 Jul 6;11(14):3938. doi: 10.3390/jcm11143938 (PMC9316533; doi:10.3390/jcm11143938)

<u>Supplementary Material</u>

Table S1 Reasons for study participation ineligibility 2
Table S2 Visual Analogue Scale (VAS) score for tolerability in intervention participants 3
Table S3. Rectus femoris cross sectional area and echogenicity in control and intervention participants 4
Table S4 : Strength and physical function measures in control and intervention participants 5
Table S5 Superficial femoral artery outcome measures in control and intervention participants 6
Table S6 Vascular and Inflammatory biomarkers levels 7

Figure S1 Sample size for outcome measures at each time point during the study 9

Table S1 Reasons for study participation ineligibility

RSCH Site		ASPH Site	
Reasons	N (%)	Reasons	N (%)
Profound coagulopathy (Prothrombin time>2.5x normal, APTT > x2 normal or platelet count of <50) or bleeding diathesis or on IV heparin infusion APTR >2	16(14.8)	Patient hospitalised > 48 hours prior to ICU admission	27(32.9)
Poor Prognosis	15(13.9)	Neuromuscular condition – any previous or concurrent neurological condition or muscle disease	20(24.4)
ICU admission > 48 hours prior	14(13.0)	History of peripheral arterial vascular disease – any previous surgery or interventional procedure for peripheral arterial insufficiency, or any reason to clinically suspect arterial insufficiency of the leg – such as a collateral history of claudication or examination findings of absent peripheral pulses	11(13.4)
Disseminated malignancy	13(12.0)	Previous, or current, DVT and PE	6(7.3)
Neuromuscular condition – any previous or concurrent neurological condition or muscle disease	9(8.3)	Disseminated malignancy	5(6.1)
Previous, or current, DVT and PE	9(8.3)	Prior amputation of a lower limb	3(3.7)
Profound cardiovascular instability – infused vasopressors ≥0.5mcg/kg/min of norepinephrine; or in the opinion of a senior attending doctor	8(7.4)	Profound cardiovascular instability – infused vasopressors ≥0.5mcg/kg/min of norepinephrine; or in the opinion of a senior attending doctor	2(2.4)
History of peripheral arterial vascular disease – any previous surgery or interventional procedure for peripheral arterial insufficiency, or any reason to clinically suspect arterial insufficiency of the leg – such as a collateral history of claudication or examination findings of absent peripheral pulses	7(6.5)	Frail skin or skin condition or soft tissue infection or other reasons that prevent access to upper limb	2(2.4)
Patient hospitalised > 48 hours prior to ICU admission	6(5.6)	Likely to remain on ICU for at least 4 days	2(2.4)
Frail skin or skin condition or soft tissue infection or other reasons that prevent access to upper limb	4(3.7)	Profound coagulopathy (Prothrombin time>2.5x normal, APTT > x2 normal or platelet count of <50) or bleeding diathesis or on IV heparin infusion APTR >2	1(1.2)
Contraindication to pharmacological venous thromboembolism prophylaxis	2(1.9)	ICU admission > 48 hours prior	1(1.2)
BMI >40/ Thigh Circumference > 77 cm	2(1.9)	Poor Prognosis	1(1.2)
Positioned prone	1(0.9)	Pre-existing significant cognitive impairment	1(1.2)
Pre-existing significant cognitive impairment	1(0.9)		

The frequency (percentage) of occurrence of each criterion in descending order. Because patients could be ineligible due to multiple factors, the sum of the frequency exceeds the total number of ineligible patients.
APTR Activated Partial Thromboplastin Time Ratio, *APTT* Activated Partial Thromboplastin Time, *BMI* Body Mass Index, *DVT* Deep Vein Thrombosis, *PE* Pulmonary Embolism

Table S2 Visual Analogue Scale (VAS) score for tolerability in intervention participants

Day	Sessions	*Participant 03*	*Participant 04*	*Participant 05*	*Participant 08*	*Participant 025*	*Participant 026*	Mean (± SD)
Day 1	1	*Sedated*	*Sedated*	-	-	*Sedated*	*Not performed*	-
	2	*Sedated*	*Sedated*	*Declined*	*Sedated*	*Not performed*	*Sedated*	-
Day 2	1	*Sedated*	*Sedated*	*Declined*	*Sedated*	*Sedated*	*Not performed*	-
	2	***8*** (-)	***6*** *(-, 130)*	*Declined*	*Sedated*	*Sedated*	*Declined*	*7 (± 1)*
Day 3	*1*	***9*** *(-, 110)*	*Not performed*	*Declined*	*Sedated*	*Sedated*	***6*** *(-)*	*8 (± 2)*
	2	*Declined*	*Not performed*	*Declined*	*Sedated*	*Sedated*	***10*** *(-)*	*10*
Day 4	*1*	*Declined*	***2*** *(-, 120)*	*Withdrawn*	*Sedated*	*Sedated*	***6*** *(-)*	*4 (± 3)*
	2	*Declined*	*Not performed*		*Sedated*	*Sedated*	***6*** *(-)*	*6*
Day 5	*1*	*Declined*	***0*** *(-, 120)*		*Sedated*	*Sedated*	***6*** *(-)*	*3 (± 4)*
	2	***0*** *(-, 95)*	***6*** *(-, 120)*		*Sedated*	*Sedated*	***6*** *(-)*	*4 (± 3)*
Day 6	*1*	***2*** *(-, 95)*	***0*** *(+, 130)*		*Sedated*	*Sedated*	*Discharged*	*1 (± 1)*
	2	***0*** *(-, 95)*	*Drowsy (125)*		*Sedated*	*Sedated*		*0*
Day 7	*1*	***2*** *(+, 95)*	*Not performed*		*Sedated*	*Sedated*		*2*
	2	***2*** *(+, 95)*	*Sedated*		*Sedated*	*Sedated*		*2*
Day 8	*1*	*Discharged*	*Sedated*		*Sedated*	*Sedated*		-
	2		*Sedated*		*Sedated*	*Sedated*		-
Day 9	*1*		*Sedated*		*Sedated*	*Sedated*		-
	2		*Sedated*		*Sedated*	*Sedated*		-
Day 10	*1*		*Sedated*		*Sedated*	*Not performed*		-
	2		*Sedated*		*Sedated*	***2*** *(+)*		*2*

Table presents VAS score on a scale of 0 (no pain) to 10 (worst possible, unbearable excruciating pain). -/+ represent if delirium present (+) or not (-) assessed by Confusion Assessment Method for the ICU (CAM-ICU). Number in bracket represent the cuff pressure in mmHg used, if protocol of cuff pressure of 50 > systolic blood pressure was deviated due to intolerability

Table S3. Rectus femoris cross sectional area and echogenicity in control and intervention participants

Outcome Measures	Day 6		Statistical Analysis
	Control (n = 5)	*Intervention* (n = 4)	
RFCSA (%)	*-14.6 (- 26.3 to - 4.3)*	*-17.4 (-18.5 to - 4.4)*	*Time p ≤ 0.05**
SFT corrected RF Echogenicity (%)	*6.8 (-0.4 to 23.4)*	*22.1 (13.5 to 29.7)*	*Time p ≤ 0.05**
Uncorrected RF Echogenicity (%)	*2.2 (-10.7 to 17.2)*	*- 0.7 (- 7.0 to 30.2)*	*Time p ≥ 0.05*
	Untreated leg (n = 3)	*Treated leg* (n = 3)	
RFCSA (%)	*-14.0 (-15.9 to -0.0)*	*-18.0 (-18.7 to -16.8)*	*Time p ≤ 0.01***
SFT corrected RF Echogenicity (%)	*19.9 (4.3 to 28.8)*	*22.1 (10.6 to 32.2)*	*Time p ≤ 0.01***
Uncorrected RF Echogenicity (%)	*-14.1 (- 21.8 to 1.1)*	*-3.3 (- 8.2 to 2.0)*	*Time p > 0.05*

Data presented as median (interquartile range) percentage (%) change from day 1 to day 6. Repeated measure data were analysed using two-way ANOVA with post hoc Bonferroni's multiple comparison test. Symbol * represent p < 0.05 and ** represent p < 0.01. *RF* Rectus Femoris; *RFCSA* Rectus femoris cross-sectional area; *SFT* subcutaneous fat thickness. Statistical analysis of between treatment groups comparison not presented as sample size underpowered.

Table S4 : Strength and physical function measures in control and intervention participants

Outcome Measures	Day 6		ICU discharge		Hospital discharge		Statistical Analysis
	Control	*Intervention*	*Control*	*Intervention*	*Control*	*Intervention*	
Strength Measures	n = 4	n = 2	n = 4	n = 2	n = 4	n = 3	
MRC-SS (score on scale of 60)	*43 (34- 53)*	*43 (42-44)*	*46 (36-48)*	*53 (50-56)*	*56 (54-59)*	*56 (50-58)*	*Time p = 0.051*
Handgrip (Kg)	*14.8 (6.4-22.1)*	*11.7 (9.6- 13.8)*	*10.2 (7.2-11.8)*	*14.1 (12.3-15.8)*	*16.7 (12.7-24.2)*	*16.9 (13.5-17.0)*	*Time p > 0.05*
Physical Function Measures	n = 4	n = 4	n = 4	n = 2	n = 4	n = 3	
ICU Mobility score (score out of 10)	*2 (1- 7)*	*3 (0- 6)*	*5 (3-7)*	*8 (8-8)*	*10 (9-10)*	*9 (8-10)*	*Time p < 0.01***
Sit to Stand (number)					*8 (4-11)*	*7 (0-7)*	
Timed up and go (seconds)					*19 (8-34)*	*15 (0-20)*	

Data presented as median (interquartile range). Day 6 sample size is lower for strength measure outcomes than ICU mobility score as only includes awake sedation free participants. Repeated measure data analysed using mixed-effect model analysis with post hoc Bonferroni's multiple comparison test. Symbol ** represent $p < 0.001$. MRC-SS Medical Research Council Sum Score. Statistical analysis of between treatment groups comparison not presented as sample size underpowered.

Table S5 Superficial femoral artery outcome measures in control and intervention participants

	Day 1		Day 6		Statistical Analysis
Outcome	*Control*	*Intervention*	*Control*	*Intervention*	
Resting	*n = 5*	*n = 3*	*n = 5*	*n = 3*	
Diameter (mm)	*5.6 (0.4)*	*6.5 (0.9)*	*5.5 (0.4)*	*6.9 (1.0)*	*Time p > 0.05*
Blood Velocity (cm/s)	*23.2 (2.4)*	*17.2 (2.6)*	*30.0 (3.2)*	*20.4 (6.7)*	*Time p > 0.05*
Blood Flow (ml/min)	*344 (56)*	*327 (50)*	*444 (95)*	*392 (7)*	*Time p > 0.05*
SR_{AUC} ($S.10^3$)	*534(57)*	*322(106)*	*755(101)*	*278(105)*	*Time p > 0.05*
Peak	*n = 2*	*n = 2*	*n = 2*	*n = 2*	
Diameter (mm)	*5.9 (1.2)*	*7.6(0.2)*	*5.7 (1.4)*	*8.0(0.2)*	*Time p > 0.05*
Blood Velocity (cm/s)	*71.6 (9.9)*	*37.0. (5.2)*	*48.1 (1.4)*	*30.4 (8.6)*	*Time p > 0.05*
Blood Flow (ml/min)	*940 (296)*	*893 (102)*	*657 (265)*	*729 (180)*	*Time p > 0.05*
SR_{AUC} ($S.10^3$)	*3040(1307)*	*1544(127)*	*2986(2135)*	*695(340)*	*Time p > 0.05*
FMD (%)	*2.1 (0.1- 4.0)*	*3.0 (2.1-3.9)*	*3.0 (1.6- 4.3)*	*2.3 (1.4-3.2)*	*Time p > 0.05*

Normally distributed data presented as mean (SEM) and not normally data as median (interquartile range). Symbol * represent p value < 0.05. Sample size lower for peak outcome measures due to missing data because of tolerability, clinical reasons (low platelet count and hypotension), and poor-quality ultrasound clips. FMD flow mediated dilation. Statistical analysis of between treatment groups comparison not presented as sample size underpowered.

Table S6 Vascular and Inflammatory biomarkers levels

Biomarkers	Day 1		Day 6		Hospital Discharge	
	Controls (n = 5)	*Intervention (n =4)*	*Controls (n = 5)*	*Intervention (n = 4)*	*Controls (n = 2)*	*Intervention (n = 3)*
**Metabolic marker**						
IGF-1, ng/ml	*46.5 (29.5-79.1)*	*46.8 (30.4-94.1)*	*57.1 (30.4-66.0)*	*85.1 (61.1- 116.9)*	*50.9 (47.8-54.0)*	*87.5 (70.2-95.9)*
Myostatin, pg/ml	*676.5 (153.4-837.5)*	*668.6 (144.1-1014.5)*	*938.8 (760.1-1659.5)*	*693.9 (498.6-1599.5)*	*3240.4 (1419.6-5061.2)*	*1191.7 (1171.6-1854.6)*
**Vascular**						
Syndecan-1, ng/ml	*7.0 (6.1-8.8)*	*6.6 (2.9-7.2)*	*9.0 (7.0-10.0)*	*6.1 (5.1-7.4)*	*6.0 (4.0-8.0)*	*2.8(2.6-5.3)*
E- Selectin, ng/ml	*46.8 (11.4-102.8)*	*76.4 (15.5-228.3)*	*33.8 (14.3-56.8)*	*51.1(16.3-99.0)*	*30.3 (15.0-45.5)*	*32.9 (23.0-86.6)*
ICAM-1, ng/ml	*1130.0 (306.5-1570.0)*	*1320.0 (871.0-2630.0)*	*1000.0 (196.8-1680.0)*	*1240.0 (1050.0-1600.0)*	*450.1 (255.9-644.3)*	*1100.0(1030.0-1310.0)*
VCAM-1, ng/ml	*7960.4 (572.1- 10586.0)*	*3841.9(1400.0-7001.2)*	*5065.4 (1680.0-9344.4)*	*2685.0 (1520.0-5611.2)*	*2250.0 (1480.0-3020.0)*	*1580.0(1570-1600.0)*
Angiopoietin-1, pg/ml	*2026.4 (423.5-4967.5)*	*1695.6 (698.3-3111.7)*	*1323.4(433.4-6483.0)*	*1480.0 (1051.5-3376.5)*	*4780.9 (1368.9-8192.9)*	*5862.8(3350.8-6091.0)*
VEGF**,** pg/ml	*52.2 (51.5-61.5)*	*46.8(45.2-64.9)*	*53.8 (39.5-82.2)*	*35.5(28.4-66.5)*	*48.4 (41.4-55.3)*	*28.8(25.6-66.2)*
**Inflammatory**						
IL-4, pg/ml	*55.2 (37.0-72.4)*	*38.3(24.8-64.2)*	*60.0(43.0-79.2)*	*37.0(32.5-40.1)*	*37.7(35.9-39.5)*	*26.2(21.8-34.2)*
TNF**,** pg/ml	*18.9 (5.1-25.8)*	*20.3 (11.2-58.0)*	*15.4 (6.4-28.9)*	*13.9(9.4-26.0)*	*10.2 (6.6- 13.8)*	*11.7 (8.0-20.9)*
TNF Receptor II**,** ng/ml	*20.2 (13.9-11.2)*	*13.9(9.3-21.5)*	*17.2(18.4-8.3)*	*11.5(10.2-14.3)*	*9.4(8.2-12.5)*	*9.5(7.9-10.4)*
TNF Receptor I**,** ng/ml	*7.2(2.3 -13.6)*	*3.0 (2.5-9.9)*	*5.0 (3.1-19.4)*	*3.4(3.0-3.6)*	*3.8(2.1-4.9)*	*1.7(1.6-2.6)*
GM-CSF**,** pg/ml	*20.3 (5.0- 20.9)*	*18.7 (14.1-28.0)*	*15.0(5.2-18.4)*	*14.6(12.1-21.7)*	*9.9 (5.4-14.3)*	*13.1 (9.2-17.4)*
GDF-15, ng/ml	*4.6 (2.1-26.8)*	*8.4 (5.2-9.3)*	*10.9 (3.2-18.5)*	*4.8 (3.6-6.0)*	*2.9 (1.5-4.3)*	*1.9(1.5-2.9)*

IL-1β, pg/ml	*16.5 (5.1-22.4)*	*18.3 (7.2-27.0)*	*15.0 (4.9-28.0)*	*13.5(6.9-19.7)*	*9.5(5.7-13.4)*	*10.7(8.3-18.6)*
IL-6, pg/ml	*38.7(16.8-369.0)*	*89.8(44.1-211.9)*	*37.2(5.1-252.3)*	*11.4(4.8-54.8)*	*13.0* (12.1-14.0)	*7.3(4.9-16.0)*
Procalcitonin, pg/ml	*720.6 (266.0-2501.7)*	*702.2(640.4-763.9)*	*558.2(96.8-2730.1)*	*218.9(111.6-1096.8)*	*80.1 (47.8-112.4)*	*80.2(59.0-91.7)*
IL-8, pg/ml	*20.5 (2.8-111.8)*	*18.6 (7.9-22.4)*	*36.7 (4.6-393.0)*	*8.2 (7.0-9.4)*	*< 3.62*	*< 3.62*

Table presents median (range) biomarkers levels in circulation during the study participants in control and intervention group. GDF-15, GM-CSF Granulocyte-macrophage colony-stimulating factor; ICAM-1 Intercellular Adhesion Molecule 1, IGF-1 Insulin-like growth factor 1, IL Interleukin; TNF Tumour Necrosis Factor; VCAM-1 Vascular cell adhesion molecule; VEGF Vascular endothelial growth factor

**IL-10 levels were below the lower value (4.03 pg/ml) of standard curve range and could not be measure*

Figure S1 Sample size for outcome measures at each time point during the study

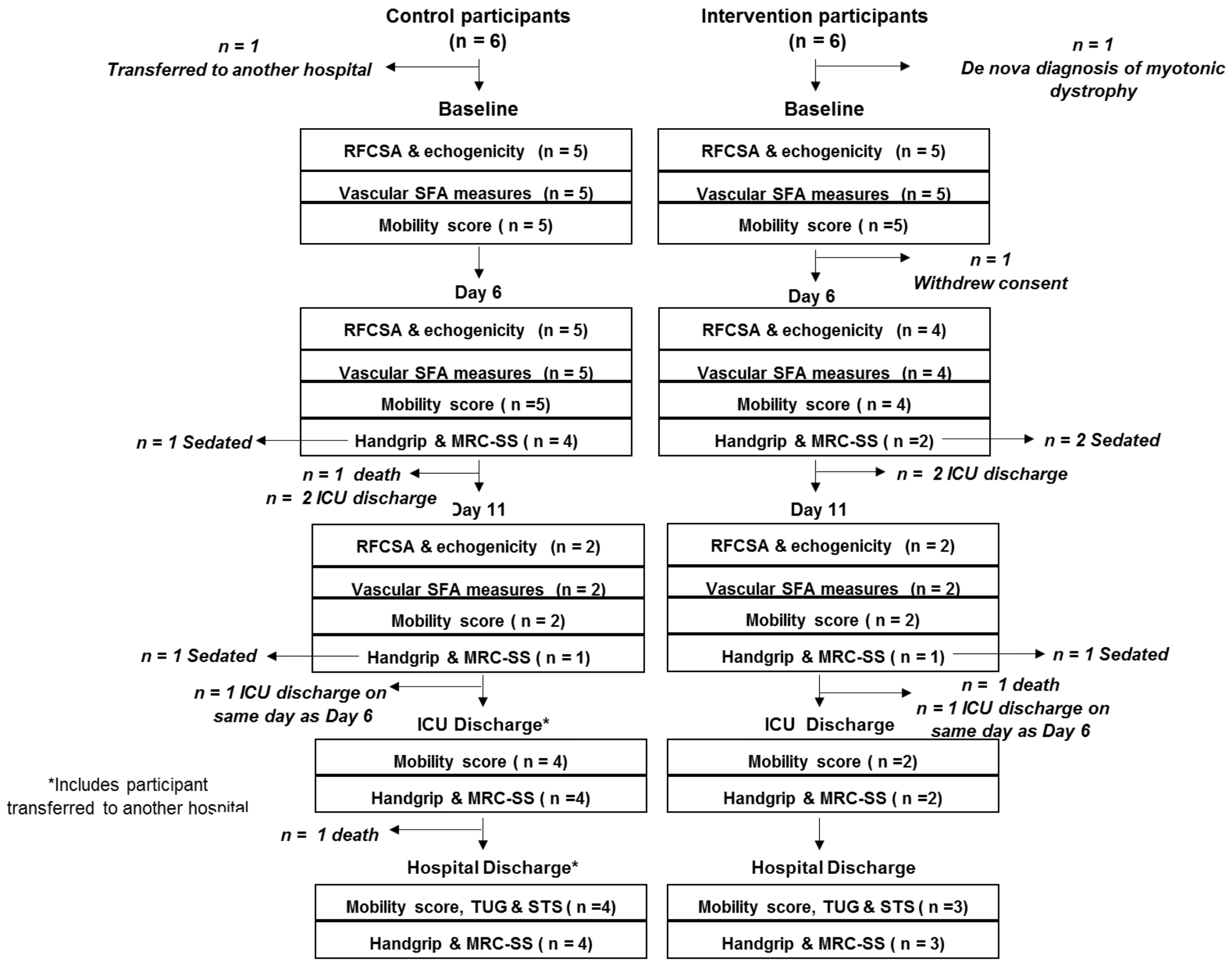

Supplement: Supplementary file 1 [file jcm-11-03938-s001.zip › ROSProx_Supplementary Material_.pdf]
